# Bioactive Egg Components and Inflammation

**DOI:** 10.3390/nu7095372

**Published:** 2015-09-16

**Authors:** Catherine J. Andersen

**Affiliations:** Department of Biology, Fairfield University, Fairfield, CT 06824, USA; E-Mail: candersen@fairfield.edu; Tel.: +1-203-254-4000 (ext. 2266); Fax: +1-203-254-4253

**Keywords:** eggs, inflammation, phospholipids, cholesterol, lutein, bioactive proteins, healthy adults, metabolic syndrome, type 2 diabetes mellitus

## Abstract

Inflammation is a normal acute response of the immune system to pathogens and tissue injury. However, chronic inflammation is known to play a significant role in the pathophysiology of numerous chronic diseases, such as cardiovascular disease, type 2 diabetes mellitus, and cancer. Thus, the impact of dietary factors on inflammation may provide key insight into mitigating chronic disease risk. Eggs are recognized as a functional food that contain a variety of bioactive compounds that can influence pro- and anti-inflammatory pathways. Interestingly, the effects of egg consumption on inflammation varies across different populations, including those that are classified as healthy, overweight, metabolic syndrome, and type 2 diabetic. The following review will discuss the pro- and anti-inflammatory properties of egg components, with a focus on egg phospholipids, cholesterol, the carotenoids lutein and zeaxanthin, and bioactive proteins. The effects of egg consumption of inflammation across human populations will additionally be presented. Together, these findings have implications for population-specific dietary recommendations and chronic disease risk.

## 1. Introduction

Inflammation is a normal, adaptive physiological response to pathogenic insult, including microbial infection and tissue injury; however the incidence of chronic low-grade, systemic inflammation underlying multiple highly prevalent chronic metabolic diseases has warranted the evaluation of inflammatory processes in disease pathogenesis [1,2]. Acute inflammatory responses mediated by immune system cells are considered beneficial if executed in a local, controlled manner, as they function to rapidly and effectively eliminate pathogenic stimuli and return the affected tissue to a normal, homeostatic state through coordinated activation and resolution of pro-inflammatory leukocyte activity [1]. However, failure of the body to appropriately execute and resolve acute inflammatory responses can lead to a detrimental chronic inflammatory tissue state, characterized by pathological tissue remodeling, fibrosis, and impaired functioning due to persistent inflammatory cell infiltration, activation, and leukocyte-mediated tissue damage [3,4]. These detrimental effects are observed in cases of inappropriate activation of the immune system, such as autoimmune conditions and allergic responses [5,6,7]. It has additionally been well established that similar adverse physiological adaptations occur in obesity-related disorders, where prolonged metabolic stress and tissue malfunction play a role in the development of chronic diseases, such as metabolic syndrome, cardiovascular disease (CVD), type 2 diabetes mellitus (T2DM), and cancer—all of which coincide with a chronic state of systemic, low-grade inflammation [8,9,10,11].

Given its significant role in the pathophysiology of many chronic diseases, inflammation has become a primary target for nutritional intervention. Various dietary patterns, functional foods, nutrients, and bioactive components have been shown to modulate inflammatory processes within the context of disease risk and progression [12,13,14,15]. Within this category, eggs are one of the most complex and controversial foods [16]. Eggs contain a variety of essential nutrients and bioactive components, but are most often recognized as a relatively rich source of high-quality protein and dietary cholesterol [17,18,19]. This had led to discrepancy in dietary recommendations across populations, where egg consumption has traditionally been considered more advisable to young, healthy populations and/or athletes (e.g., those with greater protein needs that can withstand a dietary cholesterol “challenge”), whereas egg intake by individuals at risk for CVD has been discouraged [20,21]. These recommendations have held, despite numerous epidemiological studies finding no association between egg intake and risk of coronary heart disease (CHD) mortality or stroke in the general U.S. population [22,23,24,25]. However, some studies have found eggs to be associated with an increased risk for T2DM [26,27], although these findings are not consistent across studies [28,29]. While the majority of egg nutrition studies have focused on parameters of lipid metabolism and markers of CVD risk, research has additionally revealed differential inflammatory responses across human populations. These findings indicate that healthy individuals often have greater pro-inflammatory responses to egg intake [30,31], whereas egg consumption by individuals who are overweight [32], classified with metabolic syndrome [33,34,35,36,37], or type 2 diabetic [38] is associated with a reduction in inflammatory markers. In this review, egg components known to modulate inflammatory pathways will be discussed, with a focus on their composition, bioavailability, and known mechanisms of action in cell, animal, and human models. The effects of egg consumption of inflammation across human populations will additionally be presented. Together, these findings have important implications for the role of eggs in modulating inflammation within the context of chronic diseases and immune defense.

## 2. Composition and Bioavailability of Egg Components

Eggs contain a wide variety of essential nutrients and bioactive compounds that can impact human health [17,39]. At only 72 kilocalories/large egg, eggs are a good source of high quality protein, fat-soluble and B vitamins, minerals, and choline, while providing relatively less saturated fat per gram compared to other animal protein sources [17,40]. This review will focus on primary components of eggs that are relatively abundant, bioavailable, and have pro- and anti-inflammatory properties: phospholipids, cholesterol, the carotenoids lutein and zeaxanthin, and egg white- and yolk-derived proteins.

### 2.1. Phospholipids

Eggs—particularly the yolk fractions—are one of the richest dietary sources of phospholipids [41,42]. On average, one large egg contains approximately 1.3 g of phospholipids [43,44], which represent approximately 28%–30% of total lipids by weight [45]. The predominate phospholipid class found in eggs is the glycerophospholipid phosphatidylcholine, representing approximately ~72% of phospholipids. Additional phospholipid classes include phosphatidylethanolamine (~20%), lysophosphatidylcholine (3%), phosphatidylinositol (2%), and the sphingolipid sphingomyelin (3%) [46]. While the fatty acid composition of egg phospholipids varies across classes, the majority of phospholipids contain long-chain saturated and monounsaturated fatty acids—the distribution of which can be somewhat reflective of the hen’s diet, age, and environmental conditions [44,47,48].

The majority of egg-derived phospholipids are highly bioavailable, with glycerophospholipid classes such as phosphatidylcholine being absorbed at >90% efficiency [49,50]. Tracer studies have demonstrated that dietary phospholipids are preferentially incorporated into plasma high-density lipoprotein (HDL) fractions over apoB-containing lipoproteins, red blood cells, or total blood fractions [50]. Similar findings were observed in subjects classified with metabolic syndrome, where consumption of 3 eggs per day for 12 weeks resulted in greater enrichment of HDL-phosphatidylethanolamine when compared to subjects consuming a yolk-free egg substitute. Further, subjects consuming whole eggs exhibited greater enrichment of egg-derived sphingomyelin species, indicative of the high bioavailability egg phospholipids and incorporation into HDL particles [46]. In general, phospholipids are known to influence plasma lipids and preferentially raise HDL-cholesterol [51,52], making them the likely egg component attributable to increases in HDL-cholesterol observed from egg intake [34,53].

### 2.2. Cholesterol

Eggs are one of the richest sources of dietary cholesterol, with an average large egg providing approximately 186 mg cholesterol [17]. Similar to the majority of phospholipids, cholesterol is localized to the yolk fraction; however, cholesterol only contributes ~5% of total yolk lipids by weight [45,54]. Although dietary recommendations for egg intake are often based on their cholesterol content, absorption efficiency of dietary cholesterol has been shown to be highly variable between individuals, ranging from 15% to 85% [55]. Efficiency of cholesterol absorption additionally seems to vary across human populations, with individuals who are insulin-resistant absorbing less cholesterol then insulin-sensitive counterparts [56,57], regardless of body weight status [58]. However, obese and insulin resistant subjects exhibit increased rates of endogenous cholesterol synthesis, contributing to hypercholesterolemia commonly observed in these populations [56,57,58]. Obesity is additionally associated with elevated secretion of biliary cholesterol, which may compete with dietary cholesterol for micellarization and absorption [59,60,61].

In addition to body weight and health status, cholesterol absorption efficiency is affected by food matrix composition [43,58,62,63]. The absorption of egg-derived cholesterol can be altered by interactions with phospholipids, potentially altering the mobilization of cholesterol from micelles in the intestine [41,43,63]. In Sprague-Dawley rats, egg-derived phosphatidylcholine and sphingomyelin lowered the intestinal absorption of cholesterol [43,63], whereas, the addition of lysophosphatidylcholine increased cholesterol absorption [64]. Given that phosphatidylcholine represents that vast majority of egg phospholipid species [46], intestinal cholesterol-phosphatidylcholine interactions likely limit egg-derived cholesterol absorption [43,64].

Cholesterol that is absorbed is packaged into chylomicrons and HDL by the enterocyte for ultimate release into the circulation and delivery to the liver and peripheral tissues [65,66]. Interestingly, in healthy young men (age 17–22 years), consumption of 3 whole eggs + labeled tracer led to a 52% slower fractional clearance rate of ^14^C-cholesteryl ester in plasma, indicating an increased retention time in chylomicron remnants following egg consumption [67], potentially through downregulation of receptors involved in systemic cholesterol clearance [67,68]. Overall, while cholesterol intake is known to impact plasma lipid levels, it is difficult to attribute changes in plasma lipids solely to cholesterol provided in eggs, due to the similarity of effect on plasma lipids from providing whole eggs or isolated phospholipid [51,52,53].

### 2.3. Lutein and Zeaxanthin

In addition to phospholipids and cholesterol, egg yolks contain various antioxidant carotenoids [69]. Carotenoids are plant-derived pigments that confer yellow, orange, and red color to fruits and vegetables [70]. As such, the carotenoid composition of egg yolk is reflective of the hen’s diet, with greater intake of carotenoid-rich grains resulting in greater yolk enrichment [71]. Lutein and zeaxanthin are the predominant carotenoid species found in egg yolk, although β-carotene, α-carotene, and β-cryptoxanthin are also present at lower levels [69].

Lutein and zeaxanthin are dipolar xanthophylls comprised of hydrophilic ionone ring structures with hydroxyl groups on each end, connected by a lipophilic central chain consisting of conjugated C = C bonds. Lutein and zeaxanthin structures are near identical, apart from a difference in the positioning of a double bond the ring structure [72,73]. The predominant isomers of carotenoids found in raw chicken eggs include all-E-lutein, all-E-zeaxanthin, 13′-Z-lutein, 13-Z-zeaxanthin [74,75].

Compared to plant sources, eggs contain a relatively low amount of lutein and zeaxanthin; however, egg-derived carotenoids have been shown to be significantly more bioavailable [76]. Factors affecting the bioavailability of egg-derived lutein and zeaxanthin include method of cooking and the food matrix [74,75,77]. Using an *in vitro* gastrointestinal model, Nimalaratne *et al.* [75] found that the primary egg carotenoids, all-E-lutein and all-E-zeaxanthin, were highly stable during digestion, yet the method of cooking impacted carotenoid bioaccessibility—the release of the carotenoids from the whole food matrix to allow for micellarization into an absorbable form [75,77]. Boiled eggs promoted the greatest bioaccessibility, whereas scrambling had the most deleterious effect [75]. Cooking methods have additionally been shown to differentially promote the formation of Z-isomers from all-E-lutein [74], which may impact downstream micellarization and absorption [78]. Carotenoid absorption can further be influenced by phospholipid interactions in the intestine [79,80,81].

Dietary consumption of egg-derived lutein and zeaxanthin often correlates with concentrations in plasma, where carotenoids are carried by lipoproteins [69]. In a crossover study conducted in healthy men, serum lutein concentrations were increased to the greatest extent following consumption of a lutein-enriched egg, as opposed to a lutein supplement, lutein ester supplement, or spinach [76]. Plasma lutein and zeaxanthin levels have also been shown to be increased in healthy adults following consumption of one lutein- or zeaxanthin-enriched egg per day for 90 days [82], as well as hypercholesterolemic adults who consumed 1.3 egg yolks per day for 4.5 weeks [83]. Increases in plasma lutein, zeaxanthin, and β-carotene were observed in subjects with metabolic syndrome who consumed 3 eggs per day for 3 weeks. These changes corresponded to enrichment of HDL (+20%, +57%) and low-density lipoproteins (LDL) (+9%, +46%) fractions with lutein and zeaxanthin, respectively [69].

Once in circulation, lutein is preferentially localized to the retina of the eye, which has been shown to increase macular pigment density and protect against age-related macular degeneration [72,84]. In older adults (60 years +), consumption or 2 or 4 eggs per day for 5 weeks increased serum lutein and zeaxanthin, in addition to increasing macular pigment optical density [85]. Serum zeaxanthin and macular pigment density was additionally increased in adult women (age 24–59) who consumed 6 eggs/week for 12 weeks [86]. In addition to accumulating in the eye, lutein supplementation increases lutein concentrations various other tissues, including skin [87], liver [84], and adipose [88,89]. Interestingly, obesity is associated with increased carotenoid deposition in adipose and lower circulating levels of carotenoids, potentially making them less available for other tissues [90].

### 2.4. Egg Proteins

Eggs are a good source of high-quality protein that promote protein synthesis and maintenance of skeletal muscle mass [91,92,93]. On average, one large egg provides ~6.3 g protein that is rich in essential amino acids [17,94]. Eggs also contain a variety of bioactive proteins that possess antimicrobial and immunoprotective properties—that majority of which can be found in the egg white fraction [54,95,96,97]. The predominant egg white proteins that can impact inflammation include ovalbumin (54% of egg white protein by weight), ovotransferrin (12%), ovomucin (3.5%), lysozyme (3.4%), and avidin (0.5%) [54]. Egg white additionally contains ovoinhibitor, a serine proteinase inhibitor that can reduce enzymatic digestion by trypsin and chymotrypsin, and it has been demonstrated that certain egg proteins can be absorbed intact [98,99,100]. Lysozyme is absorbed intact via endocytic and paracellular transport in proximal intestine of rats [99], whereas ovalbumin is preferentially absorbed in the distal intestine via paracellular and receptor- and clatharin-mediated endocytic transport [100]. The absorption of intact egg proteins has been implicated in mediating allergic responses to egg proteins, whereas heating and digestion of egg proteins can lower allergenicity [99,100,101]. Methods of cooking and preparation of eggs may further impact overall egg protein bioavailability. Using tracer studies, cooked egg proteins have been found to be highly digestible (~91%) as compared to raw egg protein (~51%) [102].

## 3. Pro- and Anti-Inflammatory Properties of Egg Components: Mechanisms of Action

The components of eggs highlighted above each possess unique pro- and/or anti-inflammatory properties that likely contribute to the effects that egg intake has on inflammation in human populations [30,31,32,36,54,72,103]. The following section summarizes known mechanisms of action for each egg component as it relates to inflammation and human health.

### 3.1. Phospholipids

Egg-derived phospholipids have pro- and anti-inflammatory properties via both direct and indirect mechanisms. The majority of research investigating inflammatory properties of phospholipids has focused on phosphatidylcholine. In Caco-2 cells, phosphatidylcholine (200 µmol) has been shown to inhibit TNFα-induced alterations of plasma membrane architecture required for receptor-mediated signaling, activation of the pro-inflammatory mitogen-activated protein kinases (MAPKs), extracellular-signal-regulated kinase (ERK) and p38, nuclear factor κB (NF-κB) subunit translocation to the nucleus, and up-regulation of pro-inflammatory cytokines, such as tumor necrosis factor α (TNFα), interleukin (IL)-8, intercellular adhesion molecule (ICAM)-1, monocyte chemoattractant protein (MCP)-1, interferon γ-induced protein (IP)-10, and matrix metalloproteinase (MMP)-1 [104,105]. Individuals with ulcerative colitis have lower levels of phosphatidylcholine in the gastrointestinal mucus layer, and supplementation of phosphatidylcholine has positive clinical outcomes [106,107,108]. Phosphatidylcholine supplementation through diet enrichment has additionally been shown to reduce adverse leukocyte-endothelial interactions and inflammatory joint damage in a chronic murine model of rheumatoid arthritis [109]. In a rat model of neuroinflammation, oral administration of phosphatidylcholine reduced lipopolysaccharide (LPS)-induced plasma TNFα and mitigated disturbances in hippocampal neurogenesis [110].

Despite the evidence to suggest that phosphatidylcholine is anti-inflammatory, egg phospholipids have recently been implicated in the promotion of inflammation and atherosclerosis due formation of trimethylamine-*N*-oxide (TMAO) [31,111]. Production of TMAO is dependent upon intestinal microbiota-induced conversion of phosphatidylcholine to trimethylamine (TMA), followed by oxidation of TMA by hepatic flavin-containing monooxygenase 3 (FMO3). TMAO has been shown to promote atherosclerosis in animal models, whereas high levels of plasma TMAO has been associated with increased risk for major adverse cardiovascular events in a cohort of 4007 patients [31,112]. TMAO has additionally been shown to increase adipose tissue inflammation and impair glucose tolerance in mice [111]. Egg intake has also been shown to dose-dependently increase post-prandial TMAO concentrations in plasma, although large interindividual variability was observed [113]. Variation between individuals may be attributable to differences in FMO3 expression and/or intestinal microbiota composition [114]. However, intake of more than one egg per day has been associated with lower atherosclerotic burden, as determined by coronary angiography [115]. Given that numerous epidemiological studies have failed to find an association between egg intake and atherosclerosis, additional long-term studies are needed to determine whether egg-induced TMAO production has detrimental effects on inflammation and disease risk.

### 3.2. Cholesterol

Dietary cholesterol is known to be pro-atherogenic and pro-inflammatory in animal studies [116,117]; however, these studies are often not representative of egg consumption, as cholesterol is provided in high doses as an isolated form, thus failing to take into account the phospholipid matrix, realistic dose provided by eggs, and the variability in cholesterol absorption across populations [17,43,55,57]. Nevertheless, cholesterol is known to possess pro-inflammatory properties by inducing cytotoxicity in its free, unesterified form, in addition to promoting the formation of lipid rafts in plasma membranes of leukocytes, resulting in greater hypersensitivity to activation by pro-inflammatory signaling pathways [118,119]. Increased lipid raft formation has been associated with increased pro-inflammatory responses in macrophages and T lymphocytes [119,120,121,122]. In mouse models, dietary cholesterol provided by standard atherogenic diets has additionally been shown to promote aortic inflammation and the formation of macrophage foam cells—the hallmark of atherosclerosis [123,124]. In guinea pigs fed a low-carbohydrate diet, the addition of high cholesterol (0.25/100 g) increased concentrations of total and free cholesterol in the aorta and adipose tissue, while also increasing pro-inflammatory cytokine levels in adipose [125]. In line with its atheroprotective properties, HDL and its related lipid transporter, ATP-binding cassette transport A 1 (ABCA1), have been shown to exert direct and indirect anti-inflammatory activity by reducing cellular cholesterol levels, lipid raft formation, and mitigating leukocyte inflammation [120,121,126,127,128]. This may have significant implications for egg consumption, which is known to favorably modulate HDL metabolism, as discussed in greater detail below [33,34,36,46].

### 3.3. Lutein and Zeaxanthin

Despite the relatively high bioavailability of both lutein and zeaxanthin from egg yolk [69,76], lutein has gained considerably more attention due to its protective effects against age-related macular degeneration [72,129]. Supplementation with lutein alone or in combination with zeaxanthin has been shown to have anti-inflammatory effects in a variety of experimental models. The anti-inflammatory properties of lutein are thought to be related to its antioxidant activity, conferred by its conjugated C = C double bonds that can readily quench singlet oxygen species, triplet states of photoreactive molecules, and scavenge free radicals [130,131]. Lutein has been shown to protect against cisplatin-induced DNA damage, chromosome instability, and oxidative stress in mice and HepG2 human liver cells [132,133,134]. In guinea pigs fed a hypercholesterolemic diet, lutein supplementation (0.01 g/100 g) has been shown to exert anti-inflammatory effects in the liver, aorta, and eye [84,131]. Following a 12-week period, lutein treatment lowered aortic pro-inflammatory cytokines, in addition to oxidized LDL (oxLDL) in plasma and aorta. Aortic morphology further indicated protective effects of lutein against atherosclerosis [131]. Similar anti-inflammatory effects were observed in the liver, as lutein supplementation lowered the NF-κB p65 DNA binding activity compared to control animals, in addition to lowering liver TNFα protein and hepatic free cholesterol by 43% [84]. Reductions in eye TNFα and IL-1β were additionally observed in the lutein-supplemented group [84]. In apoE^−/−^ mice, combined supplementation of lutein and egg yolk reduced detrimental ultrastructural alterations of the retina, whereas egg yolk additionally reduced the degree of systemic lipid peroxidation [135].

Studies have further shown lutein to protect mice from LPS-induced lethality, while also inhibiting NF-κB-mediated pro-inflammatory gene expression induced by hydrogen peroxide [136]. Following LPS injection, dietary lutein supplementation (50 mg/kg of feed) dose-dependently reduced TNFα mRNA expression in the spleen of F-line turkeys, while increasing mRNA expression of anti-inflammatory IL-10 [137]. Interestingly, Meriwether *et al.* [138] found that the lutein status of laying hens impacted the inflammatory immune response in chick offspring, where depletion/deficiency of lutein during embryonic development and early life was associated with greater pro-inflammatory responses to LPS [138]. Lutein derivatives generated from UV-irradiation have additionally been shown to have anti-inflammatory effects via inhibition of serum TNFα and IL-6 in LPS-treated mice [139]. Lutein has additionally been shown to suppress T_h_2 lymphocyte-mediated airway inflammation in a murine model of asthma [140]. However, in a study conducted in healthy adults by Graydon *et al.* [141], lutein (10 mg/day) and zeaxanthin (5 mg/day) supplementation for 8 weeks did not affect serum ICAM-1, VCAM-1 or CRP levels [141]. These results may be indicative of a lower bioavailability of lutein and zeaxanthin from supplements, or perhaps a lack of an anti-inflammatory effect in healthy individuals who do not exhibit physiological stress and tissue dysfunction [8,76].

### 3.4. Egg Proteins

As detailed above, eggs contain a variety of bioactive proteins in the white fraction, including ovalbumin, ovotransferrin, ovomucin, lysozyme, and avidin [54]. These proteins possess antibacterial and immunoprotective properties, yet are also capable of inducing unfavorable pro-inflammatory responses in individuals allergic to egg proteins [99,100,101]. Egg white-derived lysozyme naturally exerts antimicrobial activity against Gram-positive and Gram-negative bacteria through hydrolysis of structural peptidoglycans in the bacterial cell walls, in addition to giving rise to antibacterial peptides from within its complete protein structure through enzymatic hydrolysis [142,143]. In a porcine model of dextran sodium sulfate (DSS)-induced colitis, hen egg lysozyme supplementation reduced intestinal gene expression of pro-inflammatory cytokines (TNFα, IL-6, IFNγ, IL-8, IL-17) while increasing expression of anti-inflammatory IL-4 and transforming growth factor β (TGFβ). Further, lysozyme attenuated weight loss, colonic crypt distortion, muscle wall thickening, and gastric wall permeability observed in control DSS-treated animals [97]. Ovotransferrin, an iron-binding glycoprotein with antibacterial activity, has additionally been shown reduce inflammatory colitis pathology in a DSS-induced mouse model of colitis [95,144]. Oral administration of egg ovotransferrin reduced inflammatory cytokines, while additionally mitigating clinical markers of colitis, including weight loss and histological scores of the colon [95]. Egg yolk-derived phosvitin additionally has significant bactericidal activity against *E. coli*, which was shown to be attributable to its high metal-chelating properties, in addition to its high surface activity under thermal stress [145]. Ovokinin, a biologically active peptide derivative of ovalbumin, has been shown to lower blood pressure in spontaneously hypertensive rats when provided via oral administration [146]. This phenomenon was dependent upon the presence of egg yolk phospholipids during administration, provided as either the whole egg yolk, the yolk phospholipid fraction, or isolated egg phosphatidylcholine [146].

In addition to the bioactive proteins above, utilization of immunoglobulin Y (IgY) in medicine has additionally shown promising results in promoting passive immunity against a variety of pathogens in the treatment of conditions such as colitis, influenza, and infection of *Clostridium botulinum*, *Staphylococcus aureus*, *Candida albicans*, and *Helicobacter pylori* [96,147]. In cystic fibrosis patients, daily use of a mouthwash containing IgY antibody purified from eggs of hens immunized against *Pseudomonas aeruginosa* significantly decreased *Pseudomonas aeruginosa* colonization [148]. Together, these findings highlight a unique immunomodulatory and anti-inflammatory role of egg-derived proteins.

## 4. Effects of Egg Intake on Inflammation in Human Populations

As outlined above, eggs contain a variety of bioactive components that possess pro- and/or anti-inflammatory properties. Each of these components likely contribute to the overall response observed in human subjects following egg consumption; however, evidence suggests that the effects of egg intake on inflammatory markers differs across populations, based on body weight and health status [30,31,32,33,35,36]. The following section explores these findings, with a summary of the relationship between egg intake and inflammation presented in Table 1.

**Table 1:** Effects of egg intake on inflammation in different human populations.

Population, *n*	Intervention Conditions	Effect on Inflammation	Ref.
**Healthy Adults**			
*n = 66*	4 eggs/day for 4 weeks; AHA NCEP step 1 diet	↑ serum amyloid A, CRP	[30]
*n = 40*	2-egg meal	↑ postprandial TMAO	[31]
*Young men, n = 24*	1-, 2-, or 4-egg meal	↑ *ex vivo* J774 macrophage cell free cholesterol	[149]
*Young men and women*, *n = 50*	2 eggs/day for 4 weeks	↓ AST and ALT	[150]
**Overweight**			
*Men, n = 28*	3 eggs/day for 12 weeks, *ad libitum* carbohydrate-restricted diet	↓ CRP ↑ adiponectin	[32]
**Insulin resistant **			
*Lean, n = 76*	4 eggs/day for 4 weeks; AHA NCEP step 1 diet	↔serum amyloid A, CRP	[30]
*Obese, n = 59*	4 eggs/day for 4 weeks; AHA NCEP step 1 diet	↔serum amyloid A, CRP	[30]
**Metabolic syndrome **			
*Men and women, n = 37*	3 eggs/day for 12 weeks, moderate carbohydrate-restricted diet	↓oxidized LDL	[34]
*Men and women, n = 37*	3 eggs/day for 12 weeks, moderate carbohydrate-restricted diet	↓TNFα, serum amyloid A	[35]
*Men and women, n = 5*	3 eggs/day for 12 weeks, moderate carbohydrate-restricted diet	↓LPS-induced TNFα and IL-1β production from PBMCs *ex vivo*	[36]
**T2DM**			
*Men and women, n = 29*	1 egg/day for 5 weeks	↓TNFα and AST ↔ CRP	[38]
*Men and women, n = 65*	2 eggs/day for 12 weeks	↔CRP and homocysteine	[151]

Abbreviations: ↑: increase; ↓: decrease; ↔: no change; AHA: American Heart Association; ALT: alanine aminotransferase; AST: aspartate aminotransferase; CRP: C-reactive protein; IL-1β: interleukin 1 β; NCEP: National Cholesterol Education Program; PBMC; peripheral blood mononuclear cells; T2DM: type 2 diabetes mellitus; TMAO: trimethylamine-*N*-oxide; TNFα: tumor necrosis factor α.

### 4.1. Healthy Populations

A number of intervention trials conducted in healthy adults have demonstrated a pro-inflammatory response to egg intake. Tannock *et al.* [30] investigated the effects of egg consumption in lean insulin sensitive, lean insulin resistant, and obese insulin resistant subjects on an American Heart Association (AHA)—National Cholesterol Education Program (NCEP) step 1 diet. Interestingly, after consuming 4 eggs per day for 4 weeks, CRP and serum amyloid A—both acute phase inflammatory proteins—were significantly increased in the lean insulin sensitive subjects, whereas no changes were observed in either lean or obese insulin resistant groups—despite their having higher baseline levels of inflammation [30]. This was associated with increased cholesterol absorption in the lean insulin sensitive subjects, whereas both lean and obese insulin resistant subjects exhibited greater cholesterol synthesis [58]. In a crossover study conducted in 13 subjects with LDL-C over 130 mg/dL (>3.36 mmol/L), addition of daily egg yolk to a diet of 30% fat (predominantly polyunsaturated and saturated fatty acids) for 32 days resulted in increased susceptibility of LDL to *in vitro* oxidation [152]. Increased susceptibility to plasma and LDL oxidation was additionally observed by Levy *et al.* [153] in subjects consuming 2 eggs per day for 3 weeks. These subjects additionally exhibited minor increases in plasma glucose [153], contributing to the controversial body of research regarding the effects of egg intake on T2DM risk [26,27]. Similarly, healthy subjects who consumed a 2-egg meal exhibited increased plasma levels of pro-inflammatory TMAO postprandially; however, these increases were dependent upon the presence of normal intestinal microbiota, as administration of an oral broad-spectrum antibiotic suppressed the egg-induced increase in TMAO [31]. In a study by Ginsberg *et al.* [149], the serum from healthy men following consumption of a meal containing 0, 1, 2, or 4 eggs was incubated with J774 murine macrophage cells for 18 h. Following incubation, cellular free cholesterol content of J774 cells was highest when incubated with serum post-egg consumption when compared to the 0-egg meal [149]. Although markers of inflammation were not assessed, elevated levels of leukocyte cholesterol is known to increase the pro-inflammatory potential of the cell [120,121]. Conversely, in a study in college-aged men and women participating a crossover study, liver enzymes aspartate aminotransferase (AST) and alanine aminotransferase (ALT) were lower following consumption of a 2-egg per day for 4 weeks *vs.* an oatmeal breakfast, whereas no changes were observed in CRP [150].

### 4.2. Overweight

It has been well established that excessive weight gain and obesity is associated with a chronic state of low-grade systemic inflammation and metabolic tissue dysfunction. This physiological milieu is thought to stem from dysfunctional adipose, which becomes stressed as it attempts to expand in order to accommodate an excess influx of nutrients [8]. In contrast to what is observed in most healthy populations, egg consumption in overweight populations shows beneficial anti-inflammatory effects. In a study by Ratliff *et al.* [32], overweight men consuming 3 eggs per day for 12 weeks while following an *ad libitum* carbohydrate-restricted diet showed reductions in plasma CRP, that were not observed in overweight men consuming a carbohydrate restriction diet with yolk-free egg substitute. However, men consuming the egg substitute showed significant decreases in pro-inflammatory MCP-1 [32]. Interestingly, men on both whole egg and egg substitute groups increased plasma levels of the anti-inflammatory adipokine adiponectin over 12 weeks, with greater increases observed in the whole egg group (+21% *vs.* +7%) [32]. Consumption of eggs for breakfast has additionally been shown to increased satiety in overweight/obese women [154] and healthy men [155] when compared to a bagel breakfast, while also promoting weight loss and reductions in daily caloric intake [155,156]. Increased satiety from egg consumption has also been observed in young adults [157]. Together, these findings suggest that egg consumption may improve inflammation in overweight/obese individuals undergoing weight loss—either through direct action of bioactive components or indirect action of promoting satiety, weight loss, and restoration of adipose tissue function.

### 4.3. Metabolic Syndrome

Metabolic syndrome is characterized by a clustering of cardiometabolic risk factors that increase an individual’s risk of developing CVD and T2DM by 2- and 5-fold, respectively [20]. Individuals with metabolic syndrome commonly present with insulin resistance, endothelial dysfunction, adverse lipoprotein profiles, and a chronic state of low-grade inflammation [158]. However, similar to what has been observed in obesity, egg consumption has been shown to mitigate inflammation in metabolic syndrome. In men and women classified with metabolic syndrome following a moderate carbohydrate-restricted diet, consumption of either 3 eggs per day or the equivalent amount of yolk-free egg substitute for 12 weeks lowered oxLDL [34]. Interestingly, reductions in plasma TNFα and serum amyloid A were only observed in the group consuming whole eggs that included the yolk, whereas no changes in CRP, adiponectin, IL-6, or IL-10 were observed in either whole egg or egg substitute groups [35].

The effects of egg consumption during carbohydrate restriction in metabolic syndrome was further assessed in regard to peripheral blood mononuclear cell inflammation [33,36]. Despite increases in peripheral blood mononuclear cell (PBMC) toll-like receptor 4 (TLR4) mRNA expression, whole egg intake did not alter lipopolysaccharide-induced TNFα or IL-1β secretion by PBMCs. Surprisingly, lipopolysaccharide-induced TNFα or IL-1β secretion in PBMC was increased over the 12 week period in subjects consuming the yolk-free egg substitute. Interestingly, there was a trend toward a decrease in PBMC cholesterol content in the whole egg group, as changes in PBMC cholesterol content over the 12-week intervention positively correlated with lipid raft content [33,36]. These changes corresponded to increased PBMC mRNA expression of ABCA1, which is known to exert direct and indirect anti-inflammatory activity [120,121]. Egg consumption in metabolic syndrome has additionally been shown to increase HDL-phosphatidylethanolamine content and the *ex vivo* cholesterol-accepting capacity of serum from lipid-loaded macrophages [46]. While the anti-inflammatory properties of egg-induced, phosphatidylethanolamine-enriched HDL were not assessed, phosphatidylethanolamine may confer antithrombotic properties [159,160]. Thus, taken together with the reductions in serum amyloid A, which is predominantly associated with HDL in circulation, it is possible that some of these observations may be attributable to more anti-inflammatory and functional HDL [35,46,161].

### 4.4. T2DM

Of all populations, the recommendation of egg intake in T2DM is one of the most controversial, given the results of some epidemiological studies that found a positive association between egg intake and T2DM risk [26,27]. However, similar to what has been observed in obese and metabolic syndrome populations, egg intake in T2DM appears to reduce markers of inflammation. In a randomized, crossover study conducted in patients with well-controlled T2DM, intake of 1 whole egg per day breakfast for 5 weeks significantly reduced AST and TNFα when compared to an oatmeal-based breakfast [38]. Further, there were no differences in fasting glucose, glycosylated hemoglobin (HbA1c), CRP, or plasma lipids between the egg and oatmeal breakfast periods, suggesting that consumption of one egg per day may mitigate the inflammation characteristic of T2DM without negatively affecting traditional markers of glucose tolerance and CVD risk [38]. Similarly, in a study by Pearce *et al.* [151], T2DM patients fed a hypoenergetic high-protein, high-cholesterol diet (achieved by consuming 2 eggs/day) for 12 weeks exhibited no adverse changes in T2DM and CVD biomarkers. Conversely, egg consumption resulted in greater increases in serum HDL-cholesterol and plasma lutein when compared to T2DM patients consuming a low cholesterol hypoenergetic, high-protein diet that lacked eggs. However, no changes in serum CRP or plasma homocysteine were observed in either group [151]. Thus, it appears eggs may confer anti-inflammatory benefits in patients with well-controlled T2DM.

### 4.5. Acute Infection

The findings on egg intake and inflammation outlined above not only have implications for chronic metabolic diseases, but also for immune function in cases of acute infection, where inflammation is essential to clearing pathogenic factors. While research on egg intake in immunity is limited, one study by Pérez-Guzmán *et al.* [162] investigated the effects of a cholesterol-rich diet on the treatment of, and recovery from, pulmonary tuberculosis. Adult patients with newly diagnosed pulmonary tuberculosis were assigned to consume a cholesterol-rich diet (800 mg cholesterol/day, provided by egg yolk, butter, beef liver, and dairy products) or a control diet (250 mg cholesterol/day) for 8 weeks while remaining hospitalized and receiving anti-tubercular drug treatments. Interestingly, subjects following the cholesterol-rich diet exhibited faster reductions in sputum production and clearance of mycobacteria from sputum cultures [162]. Given these findings, and those highlighted above, the effects of egg intake in parameters of immunity across different populations warrants further investigation.

### 4.6. Implications from Human Studies

As presented above, the majority of research suggests that egg intake promotes a pro-inflammatory response in healthy adults [30,31], whereas the consumption of eggs in conditions of overweight [32], insulin resistance [30], metabolic syndrome [35,36], and T2DM [38,151] have either an anti-inflammatory or neutral effect. It is possible that this variation is attributable to differences in intestinal absorption of dietary cholesterol, which is known to be increased in healthy, non-insulin resistant individuals [30,56,57,58]; however, it is possible that other factors impact the dietary response to eggs, such as the composition of the microbiome or genetic variation [31,114]. It is additionally important to recognize potential confounding variables across studies, such as differences in the number of eggs consumed per day, concurrent dietary treatments/interventions, or medication regimens.

## 5. Conclusions

Bioactive egg components, including phospholipids, cholesterol, lutein, zeaxanthin, and proteins, possess a variety of pro- and/or anti-inflammatory properties, which may have important implications for the pathophysiology of numerous chronic diseases and immune responses to acute injury. The unique formulation of the egg food matrix significantly impacts the bioaccessibility and absorption of these components, allowing each bioactive component to likely contribute to the overall effects of egg intake on inflammatory processes. Thus, as opposed to solely basing dietary recommendations for egg intake on cholesterol content, it is likely more beneficial to consider the relationship between egg intake and inflammation in different populations. Moreover, given the essentiality of pro-inflammatory responses in normal immune defense against pathogens, further research into the role of egg intake on immunity is warranted. Together, the findings presented in this review have important implications for population-specific dietary recommendations that add complexity to current guidelines and standards of clinical practice.
